# Cardiovascular Risk Factors in Parents of Food-Allergic Children

**DOI:** 10.1097/MD.0000000000003156

**Published:** 2016-04-18

**Authors:** Sheila Ohlsson Walker, Guangyun Mao, Deanna Caruso, Xiumei Hong, Jacqueline A. Pongracic, Xiaobin Wang

**Affiliations:** From the Department of Population, Family and Reproductive Health, Center on the Early Life Origins of Disease, Johns Hopkins University, Bloomberg School of Public Health (SOW, GM, DC, XH, XW), Johns Hopkins University School of Education, Baltimore, MD (SOW), Institute for Interdisciplinary Salivary Bioscience Research, Arizona State University, Tempe, AZ (SOW), Department of Preventive Medicine, School of Environmental Science & Public Health, Wenzhou Medical University (GM), Center on Clinical and Epidemiological Eye Research, the Affiliated Eye Hospital of Wenzhou Medical University, Wenzhou, China (GM), and Division of Allergy and Immunology, Ann & Robert H. Lurie Children's Hospital of Chicago, Chicago, IL (JAP).

## Abstract

Previous studies suggest that chronic stress may induce immune system malfunction and a broad range of adverse health outcomes; however, the underlying pathways for this relationship are unclear.

Our study aimed to elucidate this question by examining the relationship between parental cardiovascular risk factors including systolic blood pressure (SBP), diastolic blood pressure (DBP), body mass index (BMI), and waist-to-hip ratio (WHR) and maternal psychological stress score (MPSS) relative to the severity of the child's food allergy (FA) and number of affected children.

SBP, DBP, BMI, and WHR were measured and calculated at the time of recruitment by trained nurses. MPSS was obtained based on self-report questionnaires covering lifestyle adjustments, perceived chronic stress, and quality of life. General linear models examined whether caregiver chronic stress was associated with FA.

For mothers with children under age 5 years, SBP, DBP and number of affected children had strong and graded relationships with severity of the child's FA. MPSS was also significantly and positively associated with child FA severity (*P* < 0.001). However, no relationships were found between FA severity, BMI, or WHR for either parent. This was also the case for paternal SBP, DBP, and number of affected children of any age.

There is a strong and graded link between cardiovascular risk and perceived stress in mothers of food-allergic children under age 5. Findings may have important implications for family-centered care of FA, may generalize to caregivers of children with chronic conditions, and extend the literature on allostatic load.

## INTRODUCTION

A growing literature links chronic stress with myriad adverse health outcomes over the life course.^1–4^ One potential mechanism is allostatic load (AL), a term for how “wear and tear” caused by unrelenting stress can become biologically embedded—“under the skin”—enhancing vulnerability to a range of chronic illnesses.^5,6^ Research suggests that parents of children with chronic conditions are at 2 to 3 times greater risk for chronic disease,^7,8^ and according to the Centers for Disease Control, chronic disease accounts for 7 out of 10 deaths—roughly 86% of U.S. healthcare expenditures.^9^ Within this, cardiovascular disease is the single largest expense category, accounting for about 1/3 of all deaths in 2011—and $1 of every $6 spent on healthcare.^9^ To examine this issue through a unique prism, our study explored the link between perceived stress and cardiovascular risk in parents of suspected, moderately and severely food-allergic (FA) children. FA is a life-long condition characterized by a potentially lethal immune response triggered by the ingestion of specific food proteins.^10^ At a prevalence rate of 5% to 8% in infants and children, and 1% to 4% in adults, FA has increased in the United States and abroad, becoming a significant public health issue.^10–12^

Caring for a FA child encompasses the most burdensome possible combination of sources of stress for parents of children with chronic conditions: it requires continuous parental vigilance to manage a serious condition that is invisible to the outside world; it has an unpredictable, relenting and remitting course; and it is life-threatening with no known cure.^13–16^ These factors are compounded by the ubiquitous nature of food. Moreover, the under-recognized nature of FA often means that such stressors often take place in the face of public and professional insensitivity.^17^ Prior research on parents of FA children has highlighted burdensome family-wide lifestyle adjustments, lower perceived quality of life (QoL), and greater stress stemming from logistical and emotional issues.^18–23^ No study we are aware of has examined whether perceived caregiver stress for parents of FA children is embedded at a biological level.

To address this unexplored gap in the research, we studied a large, well-phenotyped sample of parents of non-, moderately-, and severely FA children from a family-based FA study cohort enrolled in Chicago, IL. We sought to determine to what degree cardiovascular risk factors (systolic blood pressure (SBP), diastolic blood pressure (DBP), body mass index (BMI), and waist-hip-ratio) differed in parents of children across the FA spectrum; whether cardiovascular risk factors are moderated by the clinical condition of the FA child; and whether sociodemographic factors play a role. We hypothesize that perceived stress in caregivers of FA children may be linked with the aforementioned cardiovascular risk factors. Findings may have key implications for stress management related to caring for FA children, their siblings, and the family system, and may extend to caregivers of children with other chronic conditions.

## METHODS

### Study Population and Data Collection

The 1409 families included in this study were enrolled as part of a family-based food allergy (FA) study in Chicago, IL. Eligible families were those having either 1 or both parents with at least 1 biological child (ages 0–21 years) with or without FA who were willing to participate in the study. Families were recruited through general medical and allergy specialty clinics, community support groups, and advertisements in media. Information regarding specific food allergies, medical history, and home environment of each family member was collected through a structured questionnaire-based interview conducted by trained research staff. Information regarding psychological empowerment, quality of healthcare, and FA-specific QoL was collected through a structured questionnaire answered independently by each parent. The Institutional Review Board of Children's Memorial Hospital approved the study protocol. All participating families provided written informed consent.

### Total and Specific IgE Measurement

Total serum IgE, specific IgE for 9 food allergens (egg white, sesame, peanut, soy, milk, shrimp, walnut, cod fish, and wheat) were measured for each subject using Phadia Immuno CAP. The reported range for total IgE was from 2.0 to 5000 kU/L. The reported range for specific IgE was from 0.1 to 100 kUA/L, with >0.35 kUA/L considered positive. Total and specific IgE assays were prepared and performed by the Clinical Immunology Laboratory at Children's Memorial Hospital.

### Skin Prick Test

Skin prick tests were performed on all eligible participants using the Multitest II device (Lincoln Diagnostics) to 9 food allergens (milk, soy, egg white, wheat, fish mix, shellfish mix, peanut, sesame, and walnut). Any allergen with a mean wheal diameter (MWD) of at least 3 mm greater than the saline control was considered positive.

### Cardiovascular Risk Markers

Immediately after informed consent was obtained (and before blood draw and allergy skin testing) clinical measurements were obtained by trained research staff or clinic nurses trained in the study protocol. Height, weight, blood pressure, pulse, respiratory rate, and pulse oximetry were obtained using standard clinic equipment and protocols. Height and weight measurements were obtained twice and blood pressure measurements 3 times in succession by the same study personnel, and the average was used for analysis. BMI was calculated as weight (kg)/height (m^2^). Hip and waist measurements were obtained using the Gulick II Tape Measure (Country Technology) at umbilicus of waist and widest part of hips twice each in succession with the average used for analysis.

### Definition of FA Phenotypes

FA is defined as one who meets each of the following criteria, currently used in our ongoing NIAID-funded studies of FA^24^: timing of allergic reaction: onset of symptoms within 2 hours of food ingestion; clinical manifestations: a previous reaction to a food with a report of clear and objective findings of allergic symptoms in skin, pharynx, oral cavity, lower respiratory tract, and gastrointestinal tract; and evidence of positive food allergen-specific IgE (sIgE) and/or positive skin prick test, or by specific IgE above the 95% predictive level without known tolerance. Suspected FA is defined as one who has a parental report of FA but does not meet the criteria listed above. Control is defined as one who may or may not have a positive sIgE or positive skin prick test and is clinically tolerant to the food. Because of the size of this cohort, and for logistical reasons, food challenges were not feasible.

### Severity of FA

We further divided food-allergic children into 2 groups: *Mild/Moderate FA*: A subject met the FA definition above, but does not have tongue/airway, respiratory tract, or cardiovascular involvement and does not fulfill the criteria for anaphylaxis. *Severe FA*: The subject has clear and objective findings of anaphylaxis that affect the airway, respiratory tract, or cardiovascular system. We defined anaphylaxis based on published criteria put forth by a multidisciplinary group.^25^

In addition, we examined FA as a whole and by specific subgroups. Types of Food to which an individual is allergic: that is, egg white, cow's milk, and peanut. Number of Foods to which an individual is allergic: single food versus 2 foods versus 3 or more foods. Presence of Co-Morbid Allergic Diseases was based on maternal report of physician diagnosis (standardized questionnaire interview).

### Psychosocial Stress Score

The Chicago Cohort Health Services Questionnaire, which contained items related to QoL and parental burden and empowerment, was administered to all parents. Specifically, we assessed perceived stress via the Food Allergy Quality of Life Questionnaire (FAQL-PB),^26^ which was developed to measure the effect of children's FA on health-related QoL for caregivers. The FAQL-PB is comprised of 15 questions that assess high impact areas relative to FA, including social limitations, caregiver emotional burden, concern for the child's nutrition and health, and concerns related to typical childhood activities such as school and extracurricular activities. A 7-point Likert scale was used, and validation of the measure suggested strong internal and cross-sectional validity. For more details on the development of the instrument, see Cohen et al.^26^ The psychosocial stress score was calculated as the sum of the 15 questions.

### Statistical Analyses

Parental characteristics for those children with non-FA (normal control), suspected FA, mild/moderate FA, and severe FA were compared as follows. Continuous variables were descripted with median (Q1–Q3) and differences among the 4 groups were assessed by Kruskal–Wallis *H* test since their distributions were skewed. Categorical data were descripted with cases (%) and Chi-square test was used for the 4 groups’ comparison in the differences of proportion.

Generalized linear models were applied to examine the relationship of caregiver's vascular disease risks including SBP, DBP, BMI, and waist-to-hip ratio (WHR) with FA severity or the number of allergic children. The associations (odds ratios) between parental blood pressure (binary) and FA severity or the number of allergic children were explored by means of multiple logistic regression model based on multivariable analysis. Covariates included in the models were age, education level, race/ethnicity, smoking status, household income, and the severity of FA or the number of allergic children (Supplementary Table 1). The relationship of maternal chronic stress score (continuous variable) with FA severity or the number of allergic children was also estimated using generalized linear regression models adjusting for maternal factors (blood pressure, age, education level, race/ethnicity, smoking status), annual household income, the number of food-allergic children, breastfeeding or not and child age. All data management and data analysis were performed with SAS for windows version 9.4 (SAS Institute, Inc., Cary, NC) and the figures were drawn with SigmaPlot for windows version12.5 (Systat Software, Inc.). A 2-sided *P*-value ≤ 0.05 was designated as the significance level.

## RESULTS

### Population Characteristics

The present study included 1409 families with at least 1 biological child. One hundred seventy-five of them had no allergic children (normal control), 239 had children with suspected symptoms of FA (suspected FA), 425 had at least 1 diagnosed mild or moderate allergic child (moderate FA), and 570 had at least 1 child diagnosed with severe food allergies (severe FA). Maternal and paternal characteristics among the above 4 groups are presented in Table 1. Compared to others, older, white, nonsmoking, slim mothers with higher blood pressure were more likely to be in FA groups.

**TABLE 1:**
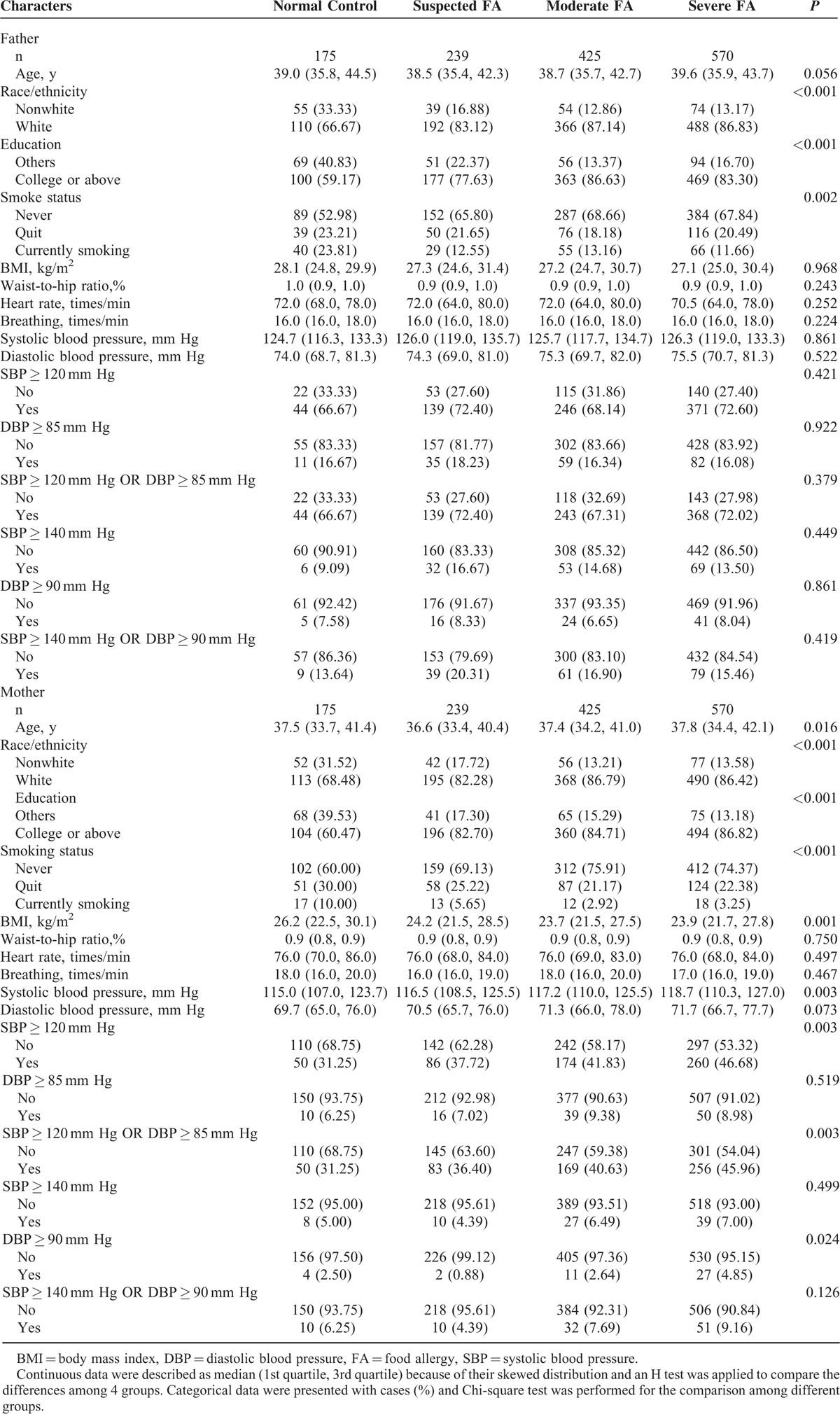
Characteristics of Fathers and Mothers

### Cardiovascular Disease Risk and FA Severity

**FIGURE 1:**
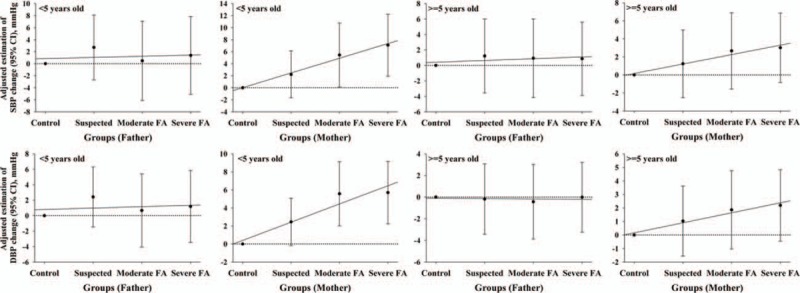
Comparison of blood pressure change between different food allergy severity groups and normal controls. FA indicates food allergy. Blood pressure change was defined as the difference between the normal control and other 3 categories of food allergy.

The mean ± standard deviation (SD) of SBP in mothers of children less than 5 years old for the normal control, suspected, moderate, and severe FA groups were 114.55 ± 12.67, 115.47 ± 11.92, 117.30 ± 11.92, and 119.17 ± 12.05 mm Hg, respectively. After adjusting for potential confounding factors, maternal SBP was observed to be significantly and positively correlated with child FA severity. Compared with normal controls, the mean estimated SBP increases were 2.21, 5.44, and 7.08 mm Hg for mothers of suspected, moderately, and severely FA children, respectively. More severe FA's were associated with higher SBP in mothers of children younger than 5 years old. A similar yet even stronger relationship between maternal DBP and child FA severity was found in the same population. However, no significant relationship between maternal SBP, DBP, and FA severity was found in mothers of children equal to and older than 5 years old. In addition, no significant relationship between paternal SBP, DBP, parents BMI or WHR and FA could be found in fathers of children of any age (Table 2, Figure 1).

**TABLE 2:**
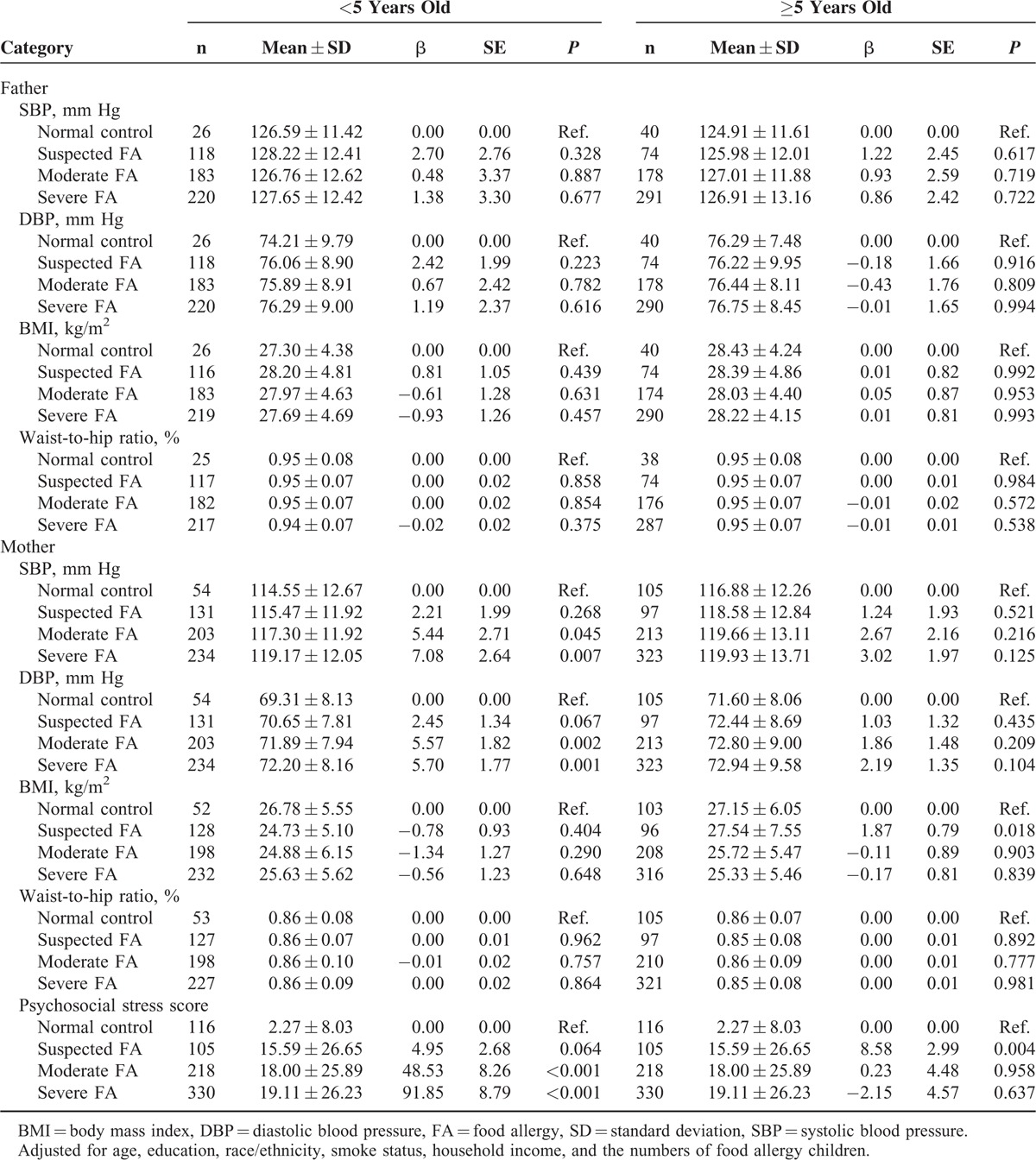
Adjusted Relationship Between Cardiovascular Disease Risk, Maternal Psychosocial Stress Score, and Severity of Food Allergy Stratified by Age of Child

### Cardiovascular Disease Risk and Number of Food-Allergic Children

In mothers of children less than 5 years old, the mean ± SD of SBP for 0, 1, and 2 or more allergic children was 115.20 ± 12.11, 118.19 ± 11.91, and 119.49 ± 13.19 mm Hg, respectively. After adjusting for potential important confounders, maternal SBP was also significantly and positively correlated with number of allergic children (*P* = 0.002 for 1 vs 0 and *P* = 0.007 for 2^+^ vs 0 allergic children). Mothers with more than one allergic child were more likely to have higher SBP and DBP in families of children less than 5 years old. However, no significant relationship between maternal SBP or DBP and the number of allergic children were observed in mothers with allergic children over or equal to 5 years old. Furthermore, no significant relationship between paternal vascular disease risk and the number of allergic children was found in families with children of any age (Table 3).

**TABLE 3:**
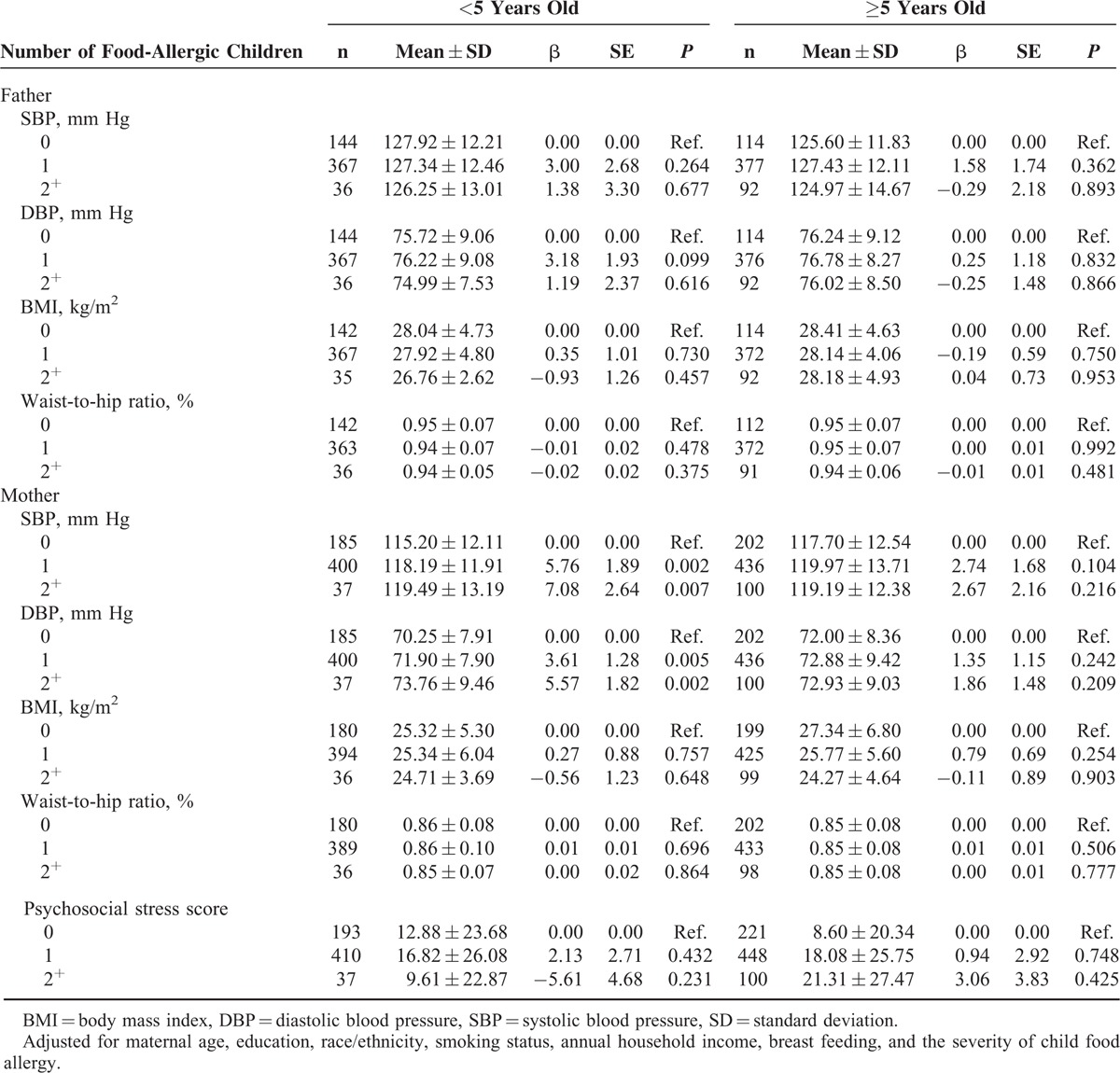
Adjusted Relationship Between Cardiovascular Disease Risk, Maternal Psychosocial Stress Score and the Number of Food-Allergic Children Stratified by Age of Child

### Maternal Perceived Stress, FA Severity, and Number of FA Children

**FIGURE 2:**
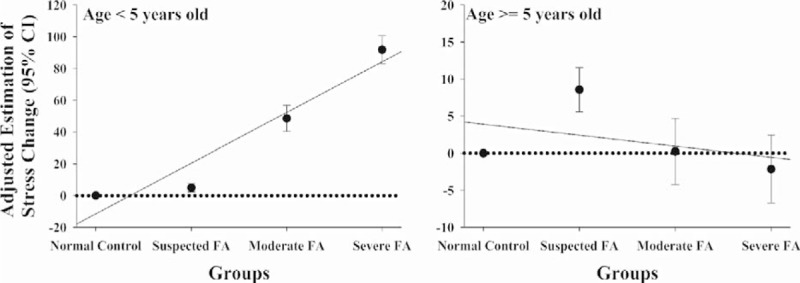
Adjusted estimation of maternal psychosocial stress score change and their 95% confidence intervals (95% CI) by severity of food allergy. The plots were derived from multivariate general linear models, and adjusted for maternal blood pressure, maternal age, maternal education level, maternal race/ethnicity, maternal smoking status, annually household income, the number of food-allergic children, breastfeeding or not and children's age.

Compared with participants in the normal control group, the average maternal perceived stress scores (MPSS) for mothers of suspected, moderately, and severely food-allergic children under 5 years old were 4.95, 45.83, and 91.85 greater than that for mothers of non-FA children, respectively. We also found that the severity of child's FA was significantly and positively correlated with increased MPSS scores. This effect was even stronger after adjusting for potential confounding risk factors. The linear trend across the 4 categories of FA severity and MPSS was significant for mothers with children less than 5 years old (*P* ≤ 0.001) (Table 2, Figure 2). However, no such relationship was found in mothers with children 5 years or older. In addition, as illustrated in Table 3, we did not find any significant relationship between MPSS and the number of FA children in all mothers, with children of any age, after adjusting for some important potential confounding factors including FA severity.

## DISCUSSION

The significant dose–response relationship found between children's FA severity, maternal perceived stress, and both SBP and DPB—each key risk factors for cardiovascular disease—provides further evidence of the important connection between psychological and physiological health. This relationship was most pronounced for mothers of children under 5 years of age—when the stress of raising a FA child may be at its greatest. SBP and DBP were elevated in a step-wise manner relative to the severity of the child's FA, and aligned with perceived stress as indexed by the MPSS. This is the first study we are aware of to examine biologically embedded cardiovascular risk factors, as well as their relationships with perceived stress, in parents of suspected, mild/moderately, and severely FA children. By studying a large, well-characterized cohort of parents under chronic stress, and examining known risk factors for cardiovascular disease, this study provides insight into the pathways by which perceived psychosocial stress “gets under the skin” and transforms into physiological symptoms.

A substantial literature exists on caregiver stress relative to dementia, chronic childhood illness, cancer, and type 2 diabetes.^27–31^ As well, recent meta-analysis affirmed the hypothesis that caregivers of children with other chronic conditions such as asthma, cancer, cystic fibrosis, diabetes, epilepsy, juvenile rheumatoid arthritis, and/or sickle cell disease experience higher levels of perceived stress.^32^ However, less is known about the psychosocial stress of caring for a FA child, and no research we are aware of investigates the mechanistic pathways that might elucidate the link between such stress and cardiovascular disease. In terms of perceived stress, the small literature in existence suggests higher perceived stress and lower QoL.^23,33–35^ These findings were validated in our study, which revealed higher levels of perceived caregiver stress by severity of the child's FA, and importantly, their age. Specifically, MPSS scores of mothers with FA children under 5 years old were 4.95, 45.83, and 91.85 greater than for non-FA children, respectively. This compares to mothers of FA children over 5, who were 8.58, 0.23, and −2.15, respectively. We believe this is because parents of younger-aged affected children must provide a comprehensive range of skills, knowledge, organizational acumen, and make significant lifestyle adjustments to properly care for the child, while simultaneously maintaining a focus on the health of not only the family, but also themselves. The internally- or externally imposed burdens requiring constant vigilance can cause substantial stress, and place an unremitting strain on the caregiver's psychological and physiological resources.

Prior studies report lower QoL for parents of FA children, stemming from issues such as significant social limitations, complications of grocery shopping and food preparation, an increased frequency of chronic and acute clinical outcomes, and a higher likelihood of other allergic diseases.^18,21^ Moreover, research suggests that caregiver stress may exceed that experienced by the FA child.^36^ The impact of caregiver stress is not limited to severe cases of FA: the QoL impact of relatively common and benign, yet chronic, gastrointestinal issues may be more taxing to caregivers than severe symptoms such as anaphylaxis and breathing difficulty, partly due to a lack of viable treatment.^20^ The bottom line is that child FA—even if moderate—can impose substantial levels of parental stress.

Our study validated previous research on parental stress, examined the issue in parents of younger and older children, and also took steps to understand how stress operates at a biological level to affect cardiovascular health risk. There are sizeable individual differences in a person's perception of stress, mediated by a combination of one's experiences, genetics, and behavior.^6,37^ This dynamic is important in light of growing research demonstrating that individual differences in perceived emotional, cognitive, and autonomic responses to stress correlate with individual differences in the stress response at a biological level.^1–3^ Equally as important is the growing body of research demonstrating that subjective stress can suppress immunological activity.^38–43^

Consistent with this premise, substantial evidence supports the damaging effects of stress on cardiovascular function.^44–49^ For example, prior research suggests that chronic stress can elevate cardiovascular risk factors in caregivers via mechanisms such as impaired endothelial function,^50^ elevated biomarkers such as tissue plasminogen activator (t-PA) antigen in caregivers relative to controls,^51^ and downstream biological effects stemming from feelings of depression and anxiety related to an individual's role as caregiver.^52,53^ Moreover, fatigued caregivers may be less likely to adhere to healthy lifestyles (e.g., nutrition, exercise, sleep, consistent social support) that can help mitigate stress, which in turn affect metabolic indicators such as BMI and waist-hip-circumference, also known as the waist-hip-ratio (WHR).^54^

Biologically mediated vulnerability to mood disorders such as anxiety or depression can also confer vulnerability in caregivers with preexisting risk,^52^ making early identification critical to prevention and intervention efforts. It is important to note that the majority of the research on cardiovascular risk in caregivers has been undertaken in elderly populations—in particular relative to Alzheimer disease and dementia. While a handful of studies have explored perceived stress, depression,^53^ and maladaptive lifestyle choices made by caregivers of children with chronic conditions, research on cardiovascular risk factors in this population is exceedingly limited. A literature search failed to identify a single study that has focused on cardiovascular risk in parents of food-allergic children.

Our study addressed this gap in the literature by examining well-accepted markers of cardiovascular risk, including SBP, DBP, WHR, and BMI in parents of non-FA, mild/moderately FA, and severely FA children. The most noteworthy result was the strong and consistent relationship between the index child's FA severity and maternal SBP and DBP. This is especially remarkable in that clinical measurements were only obtained on 1 occasion, and before other study procedures, including phlebotomy, skin testing, and questionnaires—so that results would not be affected by participant stress and apprehension. We believe that the stronger maternal (and of equal interest—not paternal) association is due to the more common role of the mother as primary caregiver. Mothers typically bear the brunt of stress around management of nutrition, social events, pediatric care, and other aspects of the child's daily life. The importance of the between-group blood pressure differential is underscored by research showing that SBP and DBP are common risk factors for hypertension, cardiovascular disease, and stroke.^55–57^

After stratifying FA children by age and controlling for parental age, we found a stronger dose–response pattern in SBP and DBP by FA severity for mothers of children under 5 years of age than for mothers of children older than 5. To reiterate, we believe that the age of the child is of tremendous relevance given the perpetually high level of vigilance required in parents of younger children stemming from their inability to adhere to strict FA food protocol. For mothers of FA children under 5 years old, the mean SBP was 118.30 mm Hg, close to the World Health Organization's SBP of 120 cut-off for high blood pressure,^58^ which automatically places the individual in a higher risk category for myriad cardiovascular-related health issues over the life course. We found no significant dose–response patterns between SBP and DBP in fathers by severity of food-allergic children of any age.

In addition to blood pressure, we examined other types metabolic data to discern whether distinct but related cardiovascular risk factors were evident in parents of FA and non-FA children. We found no significant differences in this series of analyses. BMI was similar for parents of all groups of children, as was WHR, for children of all ages. Collectively, our findings on SBP, DBP, BMI, and WHR added to the limited body of research on the biological embedding of chronic caregiver stress, with a specific focus on risk factors for cardiovascular disease.

Components of cardiovascular risk are included in a comprehensive biological metric known as “allostatic load” (AL), which was initially conceptualized to quantify the manner in which stress can become biologically embedded.^6^ AL describes the cumulative system-wide “wear and tear” on the body over time stemming from dysregulated biological concomitants of stress, and is associated with a range of adverse cognitive, physical and mental health, and QoL outcomes, as well as premature mortality.^37,59–61^ The index is comprised of a composite of cardiovascular (e.g., SBP, DBP), metabolic (e.g., WHR, BMI), the hypothalamic–pituitary–adrenal (HPA) axis (e.g., cortisol, adrenaline) and various inflammatory biomarkers (e.g., c-reactive protein, IL-6).^5,6,59^ When an individual has a specified proportion of individual biomarkers in the quartile associated with health risk, they meet the criteria for AL,^6,59,62^ placing them at significantly higher risk for long-term adverse health outcomes. While not yet a formal diagnostic criteria, the AL index operates much like the cut-off for blood pressure, as described above. The present findings infer that caregivers of FA children under 5 may be at increased risk for AL, given that high SBP and DBP increase AL scores, and higher AL scores indicate greater risk. For the caregiver, this makes a healthy lifestyle and diligent stress management habits particularly essential in order to maintain the resilience necessary for a high QoL.

Harnessing innovative biosocial research may transform how clinicians shape intervention and prevention programs, leading to innovative strategies for caregiver symptom management, ultimately disrupting the link between chronic stress and disease. Importantly, caregiver health is important for the individual, for the psychological well-being of the affected child,^63^ and also for the mental and physical health of the broader family given that individuals operate not in isolation—but in dynamic systems.^64^ Given our ever-increasing knowledge about the significant long-term health implications, our research may elucidate the need for disciplined stress management practices that enhance the health and well-being of caregivers and their families.

### Limitations

Food allergies were not confirmed by food challenge but by fulfilling accepted, previously published clinical criteria: optimizing our FA phenotype to include only those with clear objective symptoms, focusing the timing of symptoms close to ingestion, and corroborating test results. Although this is not equal to performing food challenges, we have previously corroborated diagnoses amongst a subset of study subjects with FA who have had either oral food challenges or a previous history of anaphylaxis, and have been followed in the allergy clinic at our tertiary care center.^65^

The growing recognition of the link between chronic stress and disease requires a trans-disciplinary approach to bridge the behavioral and biological sciences, further our understanding of the physiological effects of stress, and establish the foundation for a new paradigm of human health and disease. Our main conclusion is that there is a significant link between important risk factors for cardiovascular disease and perceived stress in mothers of FA children—in particular for those with FA children under 5 years old. Our findings provide strong evidence for a pathway by which chronic stress can increase vulnerability to cardiovascular disease. Findings may have important implications for family-centered care of FA, and generalize more broadly to caregivers of children with chronic conditions. Given the growing literature on the relationship perceived stress and disease, the present study underscores the need for further investigation into mechanisms by which stress may be subclinically embedded in caregivers of children with chronic conditions to enhance health and well-being for themselves, and concomitantly, their families.

## Supplementary Material

Supplemental Digital Content
