# Cardiovascular Risk Factors in Parents of Food-Allergic Children: Erratum

**DOI:** 10.1097/01.md.0000490126.65286.e6

**Published:** 2016-07-29

In the article “Cardiovascular Risk Factors in Parents of Food-Allergic Children”,^1^ which appeared in Volume 95, Issue 15 of *Medicine*, the Acknowledgments section was omitted. The article has since been corrected online.
