# Supplementary material for: Cardiovascular Risk Factors in Parents of Food-Allergic Children
Source: Medicine (Baltimore). 2016 Apr 18;95(15):e3156. doi: 10.1097/MD.0000000000003156 (PMC4839798; doi:10.1097/MD.0000000000003156)
Supplement: Supplemental Digital Content [file medi-95-e3156-s001.doc]

Supplementary Table 1. Adjusted association between the risk of high blood pressure and food allergy severity stratified by age of FA child.

| Category | <5 years old | | | |  | ≥5 years old | | | |
| --- | --- | --- | --- | --- | --- | --- | --- | --- | --- |
|  | n | cases(%) | OR(95%CI) | P |  | n | cases(%) | OR(95%CI) | P |
| Father |  |  |  |  |  |  |  |  |  |
| SBP≥120mmHg |  |  |  |  |  |  |  |  |  |
| Normal control | 26 | 18(69.20) | 1.00(1.00,1.00) | Ref. |  | 40 | 26(65.00) | 1.00(1.00,1.00) | Ref. |
| Suspected FA | 118 | 89(75.40) | 1.70(0.62,4.62) | 0.299 |  | 74 | 50(67.60) | 1.01(0.43,2.34) | 0.991 |
| Moderate FA | 183 | 121(66.10) | 0.59(0.18,1.92) | 0.382 |  | 178 | 125(70.20) | 1.26(0.59,2.73) | 0.553 |
| Severe FA | 220 | 162(73.60) | 0.89(0.28,2.82) | 0.839 |  | 291 | 209(71.80) | 1.39(0.65,2.97) | 0.394 |
| DBP≥85mmHg |  |  |  |  |  |  |  |  |  |
| Normal control | 58 | 3(5.20) | 1.00(1.00,1.00) | Ref. |  | 116 | 2(1.70) | 1.00(1.00,1.00) | Ref. |
| Suspected FA | 135 | 9(6.70) | 0.55(0.13,2.42) | 0.429 |  | 105 | 7(6.70) | 2.15(0.41,11.38) | 0.367 |
| Moderate FA | 207 | 15(7.20) | 0.32(0.04,2.46) | 0.275 |  | 218 | 9(4.10) | 1.26(0.25,6.42) | 0.777 |
| Severe FA | 240 | 19(7.90) | 0.35(0.05,2.50) | 0.293 |  | 330 | 22(6.70) | 2.05(0.44,9.58) | 0.363 |
| SBP≥120 or DBP≥85mmHg |  |  |  |  |  |  |  |  |  |
| Normal control | 58 | 5(8.60) | 1.00(1.00,1.00) | Ref. |  | 116 | 4(3.40) | 1.00(1.00,1.00) | Ref. |
| Suspected FA | 135 | 23(17.00) | 1.21(0.35,4.18) | 0.767 |  | 105 | 15(14.30) | 2.56(0.75,8.75) | 0.135 |
| Moderate FA | 207 | 31(15.00) | 1.22(0.27,5.48) | 0.800 |  | 218 | 26(11.90) | 1.91(0.59,6.16) | 0.277 |
| Severe FA | 240 | 35(14.60) | 1.11(0.25,4.89) | 0.888 |  | 330 | 43(13.00) | 1.90(0.61,5.97) | 0.271 |
| SBP≥140mmHg |  |  |  |  |  |  |  |  |  |
| Normal control | 26 | 3(11.50) | 1.00(1.00,1.00) | Ref. |  | 40 | 3(7.50) | 1.00(1.00,1.00) | Ref. |
| Suspected FA | 118 | 20(16.90) | 2.32(0.47,11.41) | 0.301 |  | 74 | 12(16.20) | 2.49(0.63,9.85) | 0.193 |
| Moderate FA | 183 | 31(16.90) | 3.03(0.50,18.32) | 0.227 |  | 178 | 22(12.40) | 1.93(0.52,7.15) | 0.323 |
| Severe FA | 220 | 32(14.50) | 2.42(0.41,14.37) | 0.332 |  | 291 | 37(12.70) | 1.94(0.54,6.98) | 0.312 |
| DBP≥90mmHg |  |  |  |  |  |  |  |  |  |
| Normal control | 26 | 3(11.50) | 1.00(1.00,1.00) | Ref. |  | 40 | 2(5.00) | 1.00(1.00,1.00) | Ref. |
| Suspected FA | 118 | 9(7.60) | 0.57(0.13,2.47) | 0.450 |  | 74 | 7(9.50) | 2.16(0.41,11.42) | 0.366 |
| Moderate FA | 183 | 15(8.20) | 0.31(0.04,2.33) | 0.254 |  | 178 | 9(5.10) | 1.28(0.25,6.50) | 0.766 |
| Severe FA | 220 | 19(8.60) | 0.33(0.05,2.35) | 0.268 |  | 290 | 22(7.60) | 2.09(0.45,9.78) | 0.351 |
| SBP≥140 or DBP≥90mmHg |  |  |  |  |  |  |  |  |  |
| Normal control | 26 | 5(19.20) | 1.00(1.00,1.00) | Ref. |  | 40 | 4(10.00) | 1.00(1.00,1.00) | Ref. |
| Suspected FA | 118 | 23(19.50) | 1.17(0.34,4.04) | 0.802 |  | 74 | 16(21.60) | 2.79(0.82,9.47) | 0.101 |
| Moderate FA | 183 | 34(18.60) | 1.20(0.27,5.37) | 0.809 |  | 178 | 27(15.20) | 2.00(0.62,6.42) | 0.244 |
| Severe FA | 220 | 36(16.40) | 1.01(0.23,4.40) | 0.995 |  | 291 | 43(14.80) | 1.89(0.60,5.92) | 0.276 |
| Mother |  |  |  |  |  |  |  |  |  |
| SBP≥120mmHg |  |  |  |  |  |  |  |  |  |
| Normal control | 54 | 13(24.10) | 1.00(1.00,1.00) | Ref. |  | 105 | 37(35.20) | 1.00(1.00,1.00) | Ref. |
| Suspected FA | 131 | 42(32.10) | 2.02(0.88,4.61) | 0.097 |  | 97 | 44(45.40) | 1.55(0.84,2.85) | 0.163 |
| Moderate FA | 203 | 81(39.90) | 2.55(0.91,7.15) | 0.074 |  | 213 | 93(43.70) | 1.57(0.92,2.70) | 0.100 |
| Severe FA | 234 | 108(46.20) | 3.25(1.19,8.90) | 0.022 |  | 323 | 152(47.10) | 1.84(1.08,3.11) | 0.024 |
| DBP≥85mmHg |  |  |  |  |  |  |  |  |  |
| Normal control | 58 | 2(3.40) | 1.00(1.00,1.00) | Ref. |  | 116 | 2(1.70) | 1.00(1.00,1.00) | Ref. |
| Suspected FA | 135 | 2(1.50) | 0.55(0.05,5.77) | 0.619 |  | 105 | 0(0.00) | 0.00(0.00,0.35) | 1.000 |
| Moderate FA | 207 | 1(0.50) | 1.00(0.05,22.58) | 0.998 |  | 218 | 10(4.60) | 3.80(0.66,21.79) | 0.135 |
| Severe FA | 240 | 7(2.90) | 5.88(0.50,69.02) | 0.159 |  | 330 | 20(6.10) | 6.37(1.12,36.12) | 0.036 |
| SBP≥120 or DBP≥85mmHg |  |  |  |  |  |  |  |  |  |
| Normal control | 58 | 4(6.90) | 1.00(1.00,1.00) | Ref. |  | 116 | 6(5.20) | 1.00(1.00,1.00) | Ref. |
| Suspected FA | 135 | 4(3.00) | 0.36(0.08,1.71) | 0.200 |  | 105 | 4(3.80) | 0.65(0.17,2.50) | 0.531 |
| Moderate FA | 207 | 9(4.30) | 0.56(0.08,4.06) | 0.569 |  | 218 | 21(9.60) | 1.89(0.68,5.26) | 0.224 |
| Severe FA | 240 | 17(7.10) | 1.03(0.16,6.69) | 0.979 |  | 330 | 34(10.30) | 2.17(0.79,5.92) | 0.131 |
| SBP≥140mmHg |  |  |  |  |  |  |  |  |  |
| Normal control | 54 | 3(5.60) | 1.00(1.00,1.00) | Ref. |  | 105 | 5(4.80) | 1.00(1.00,1.00) | Ref. |
| Suspected FA | 131 | 5(3.80) | 0.58(0.12,2.78) | 0.493 |  | 97 | 5(5.20) | 0.82(0.22,3.10) | 0.768 |
| Moderate FA | 203 | 9(4.40) | 0.78(0.10,6.07) | 0.816 |  | 213 | 18(8.50) | 1.67(0.56,5.03) | 0.361 |
| Severe FA | 234 | 15(6.40) | 1.24(0.18,8.79) | 0.831 |  | 323 | 24(7.40) | 1.37(0.46,4.04) | 0.573 |
| DBP≥90mmHg |  |  |  |  |  |  |  |  |  |
| Normal control | 54 | 2(3.70) | 1.00(1.00,1.00) | Ref. |  | 105 | 2(1.90) | 1.00(1.00,1.00) | Ref. |
| Suspected FA | 131 | 2(1.50) | 0.50(0.05,5.50) | 0.572 |  | 97 | 0(0.00) | 0.00(0.00,0.56) | 0.999 |
| Moderate FA | 203 | 1(0.50) | 1.14(0.05,26.83) | 0.933 |  | 213 | 10(4.70) | 3.80(0.66,21.83) | 0.134 |
| Severe FA | 234 | 7(3.00) | 6.01(0.49,74.35) | 0.163 |  | 323 | 20(6.20) | 6.36(1.12,36.06) | 0.037 |
| SBP≥140 or DBP≥90mmHg |  |  |  |  |  |  |  |  |  |
| Normal control | 54 | 4(7.40) | 1.00(1.00,1.00) | Ref. |  | 105 | 6(5.70) | 1.00(1.00,1.00) | Ref. |
| Suspected FA | 131 | 5(3.80) | 0.50(0.11,2.20) | 0.356 |  | 97 | 5(5.20) | 0.83(0.23,2.97) | 0.777 |
| Moderate FA | 203 | 9(4.40) | 0.60(0.08,4.36) | 0.612 |  | 213 | 23(10.80) | 2.02(0.74,5.57) | 0.172 |
| Severe FA | 234 | 17(7.30) | 1.05(0.16,6.87) | 0.962 |  | 323 | 34(10.50) | 2.05(0.75,5.59) | 0.160 |

Adjusted for age, education, race/ethnicity, smoke status, household income and the number of food allergic children.
